# Assessment of Metabolomic and Proteomic Biomarkers in Detection and Prognosis of Progression of Renal Function in Chronic Kidney Disease

**DOI:** 10.1371/journal.pone.0096955

**Published:** 2014-05-09

**Authors:** Esther Nkuipou-Kenfack, Flore Duranton, Nathalie Gayrard, Àngel Argilés, Ulrika Lundin, Klaus M. Weinberger, Mohammed Dakna, Christian Delles, William Mullen, Holger Husi, Julie Klein, Thomas Koeck, Petra Zürbig, Harald Mischak

**Affiliations:** 1 Mosaiques Diagnostics GmbH, Hannover, Germany; 2 Department of Toxicology, Hannover Medical School, Hannover, Germany; 3 RD Néphrologie, Montpellier, France; 4 Biocrates life sciences AG, Innsbruck, Austria; 5 sAnalytiCo Ltd, Belfast, United Kingdom; 6 Department of Biomedical Informatics and Mechatronics, Private University for Health Sciences, Medical Informatics and Technology, Hall in Tirol, Austria; 7 Néphrologie Dialyse St Guilhem, Séte, France; 8 Service de Néphrologie, Dialyse Péritonéale et Transplantation, Montpellier, France; 9 BHF Glasgow Cardiovascular Research Centre, University of Glasgow, Glasgow, United Kingdom; I2MC INSERM UMR U1048, France

## Abstract

Chronic kidney disease (CKD) is part of a number of systemic and renal diseases and may reach epidemic proportions over the next decade. Efforts have been made to improve diagnosis and management of CKD. We hypothesised that combining metabolomic and proteomic approaches could generate a more systemic and complete view of the disease mechanisms. To test this approach, we examined samples from a cohort of 49 patients representing different stages of CKD. Urine samples were analysed for proteomic changes using capillary electrophoresis-mass spectrometry and urine and plasma samples for metabolomic changes using different mass spectrometry-based techniques. The training set included 20 CKD patients selected according to their estimated glomerular filtration rate (eGFR) at mild (59.9±16.5 mL/min/1.73 m^2^; *n* = 10) or advanced (8.9±4.5 mL/min/1.73 m^2^; *n* = 10) CKD and the remaining 29 patients left for the test set. We identified a panel of 76 statistically significant metabolites and peptides that correlated with CKD in the training set. We combined these biomarkers in different classifiers and then performed correlation analyses with eGFR at baseline and follow-up after 2.8±0.8 years in the test set. A solely plasma metabolite biomarker-based classifier significantly correlated with the loss of kidney function in the test set at baseline and follow-up (ρ = −0.8031; p<0.0001 and ρ = −0.6009; p = 0.0019, respectively). Similarly, a urinary metabolite biomarker-based classifier did reveal significant association to kidney function (ρ = −0.6557; p = 0.0001 and ρ = −0.6574; p = 0.0005). A classifier utilising 46 identified urinary peptide biomarkers performed statistically equivalent to the urinary and plasma metabolite classifier (ρ = −0.7752; p<0.0001 and ρ = −0.8400; p<0.0001). The combination of both urinary proteomic and urinary and plasma metabolic biomarkers did not improve the correlation with eGFR. In conclusion, we found excellent association of plasma and urinary metabolites and urinary peptides with kidney function, and disease progression, but no added value in combining the different biomarkers data.

## Introduction

Chronic kidney disease (CKD) is characterised by progressive loss of renal function resulting in reduced glomerular filtration. The condition is categorised into 5 different stages with the final stage being end-stage renal failure [1]. Although current clinical analytical methods are accurate in diagnosing advanced kidney dysfunction, this is not the case for early stages [2]. Most importantly, tools for predicting the risk of progression towards end-stage renal failure are lacking and developing accurate biomarkers for prognosis of CKD progression represents a clinical challenge. Hence, efforts are directed towards earlier detection and better prognosis in order to allow for better therapeutic interventions to slow down or potentially prevent the progression of the disease in the future [3]. New technologies such as “omics”-based approaches, including proteomics and metabolomics, provide more insight into disease mechanisms and therefore hold the potential to improve management of CKD by providing stage-specific biomarkers [4]. Proteomic methods are widely used to identify biomarkers in tissues [5] and various other biological entities including urine [6]. We have recently developed a CKD classifier based on 273 urinary peptides (CKD273) with high specificity and sensitivity for the diagnosis of CKD [7]. In the course of this study 889 urine samples of healthy volunteers and patients with CKD were analysed using capillary electrophoresis–mass spectrometry (CE-MS). The CKD273 classifier performs better than the currently used markers (i.e. albuminuria and serum creatinine) in the early diagnosis of diabetic nephropathy [8], [9]. In addition, a recent study used plasma metabolomics to investigate the decline of the renal function [10] and to predict incident CKD [11]. The latter study utilised a large cohort comprising of 1434 participants and identified 16 metabolites in the plasma significantly associated with CKD via liquid chromatography-mass spectrometry (LC-MS); 9 of these metabolites performed better than serum creatinine. In a prospective cohort, a urinary metabolite-based profile was found to have diagnostic and monitoring values in CKD [12]. Proteomics and metabolomics therefore seem to enable displaying CKD stages with high confidence. However, the potential of the combination of the two technologies in improving CKD diagnosis has never been explored so far. We thus hypothesised that proteomic and metabolomic biomarkers might perform even better when combined.

In the present study, our aim was to investigate the potential value of molecular classifiers for CKD that combine urinary and/or plasma metabolomics with urinary proteomics. The study cohort comprised a group of patients from mild to moderate and advanced CKD, classified by the estimated glomerular filtration rate (eGFR). For this cohort metabolomic and proteomic data were available and allowed for a direct and uniform comparison of metabolomic and proteomic traits. Thus, we established a new proteomic CKD classifier based on this cohort and did not use the previously established proteomic classifier CKD273. We indeed established such classifiers and then tested their performance at baseline and at follow-up after 2.8±0.8 (mean ± SD) years.

## Patients and Methods

### Patients

Some of the data evaluated in this study have already been described in previous manuscripts [13], [14]. During three consecutive days, all patients eligible and attending the outpatient clinics of the hospitals of Sète and Montpellier, as well as the dialysis unit in Sète, were invited to participate in the study. Clinically stable patients, over 18 years old, who have not been admitted to hospital for at least 2 months and did not have acute inflammatory diseases, were included. A total of 49 patients were involved in the study; of those, 26 had diabetic nephropathy and the remaining 23 had other aetiologies. For evaluation, glomerular filtration rate (eGFR) was estimated by the simplified MDRD formula [15].

Plasma and urine samples were obtained from all patients. Fresh, midstream urine was collected and centrifuged; protein and creatinine concentrations were determined by the hospital laboratory. Two aliquots of urine were frozen immediately for proteomic and metabolomic analyses as described below, and stored at −80°C until analysis. Blood samples were collected in EDTA-containing tubes. Blood was put on ice and immediately centrifuged (10 minutes at 2000×g or following the tube manufacturer's instructions) at 4°C. Plasma was removed and stored at −80°C until analysis. Urine and plasma samples were coded and shipped to two laboratories. Samples were unblinded after receiving results.

The patients were subsequently seen regularly in the outpatient clinic; clinical and laboratory data were recorded. Patient management during the follow-up period was only based on usual clinical care. When patients did not attend the clinic, data were obtained from general practitioners. After 2.8±0.8 years of follow-up, outcome was obtained from 43 patients. Of those, eight patients started dialysis and four patients died not being on dialysis.

The study was designed and conducted fulfilling all the requisites of the French law on the protection of individuals collaborating in medical research and was in accordance with the principles of the Declaration of Helsinki. Written informed consent was obtained from all participants. The data were handled according to the rules of the CNIL (Centre National d'Informatique et Liberté) warranting the respect of privacy. Sample collection was declared to the French Ministry with the allocated reference number DC – 2008 – 417 and was approved by the local ethics committee, the Comité de Protection de Personnes (CPP) of Montpellier. The CPP is based in the University Hospital of Montpellier [13].

### Study design

According to current recommendations [16], [17], a training set was determined and the results were assessed in an independent test set (Table 1). The training set was selected based on eGFR measurements at baseline of the 49 samples used in this study. The “mild CKD” group was defined by patients with the highest eGFRs (59.9±16.5 mL/min/1.73 m^2^; mean ± SD) whereas the “advanced CKD” group was defined by patients with the lowest eGFRs (8.9±4.5 mL/min/1.73 m^2^; mean ± SD) (Table 1). The total sample of the training set was 20 patients equally distributed between the two groups and the remaining 29 patients constituted the independent test set in agreement with current recommendations for clinical biomarker studies [18]. However, as age and gender are two factors used to calculate eGFR, they were taken into consideration during study design and the training set was sex and age matched. A follow-up cohort was provided after 2.8±0.8 years to investigate the progression of renal function. Inter-group comparison of the mean age in the training set was achieved using t-test.

**Table 1:** Patients Characteristics.

	Training set			Test set
	“mild CKD”	“advanced CKD”	p-values	
***n***	10	10		29
**Age (years)**	65.9±10.9	70.7±9.8	0.2767	73.3±9
**Gender (M/F)**	7/3	7/3		17/12
**Baseline eGFR (mL/min/1.73 m^2^)**	59.9±16.5	8.9±4.5	<0.0001	29.5±15.6
**Follow-up eGFR (mL/min/1.73 m^2^)** *	61.2±26.2	8.7±3.1	0.0025	28.1±14.5
**BMI (kg/ m^2^)**	31.5±5.9	29±4.7	0.3085	29.7±6.7
**Serum creatinine (µmol/L)**	110.7±27.1	473.7±162.2	<0.0001	232.4±136.7
**Serum albumin (g/L)**	41.6±2.4	35.5±3.7	0.0004	38.5±3.1
**CRP (mg/L)**	3.4±3.0	4.9±4.4	0.3848	4.4±3.9

*The mean duration of the follow-up study was 2.8±0.8 years.

### Methods

#### Metabolome analysis

Targeted metabolome analysis was performed using the Absolute***IDQ*** p180 Kit (BIOCRATES Life Sciences AG, Innsbruck, Austria). The commercially available Absolute***IDQ*** p180 kits were used according to the manufacturer's instructions for the quantitation of amino acids, acylcarnitines, sphingomyelins, phosphatidylcholines, hexose (glucose), and biogenic amines. The fully automated assay was based on PITC (phenylisothiocyanate)-derivatization in the presence of isotopically labelled internal standards followed by flow injection analysis tandem mass spectrometry (FIA-MS/MS) (acylcarnitines, lipids, and hexose) as well as liquid chromatography (LC)-MS/MS (amino acids and biogenic amines). Multiple reaction monitoring (MRM) detection was used for quantitation. Prostaglandins, other oxidised polyunsaturated fatty acids and bile acids were extracted in aqueous acetonitrile containing deuterated internal standards [19]. The metabolites were determined by reverse phase HPLC-ESI-MS/MS in negative MRM detection mode. For determining reducing mono-, di- and oligosaccharides, samples were labelled with 1-phenyl-3-methyl pyrazolone in the presence of internal standards. The derivative allowed sugars to be isolated, desalted and concentrated using C18 solid-phase extraction (SPE). Sugar concentrations were determined by FIA-MS/MS using MRM mode in positive and negative ion mode. For quantitation of energy metabolism intermediates from the citrate cycle, glycolysis, pentose phosphate pathway and urea cycle in the presence of internal standards, an LC-MS/MS method in MRM mode was performed. All above described assays used an API4000 QTrap tandem mass spectrometer instrument with electrospray ionisation (AB Sciex, Concord, Canada) for quantitation. The content of free and total fatty acids was determined as their corresponding methyl ester derivatives (FAMEs) using gas chromatography (GC) coupled with mass spectrometric detection (Agilent 7890 GC/5795 MSD, Agilent Technologies, Santa Clara, CA, USA) with an electron impact ion source in SIM mode against external standards after derivatisation. Where no external standard was available, compounds were measured semi-quantitatively using spectra recorded in SCAN mode, respective ratios of characteristic ions and the retention behaviour. The (semi)-quantitation was carried out with response factors extra- and/or intrapolated from the nearby eluting compounds having the same number of double bonds.

The concentrations of amino acids, amines, eicosanoides and bile acids were calculated with Analyst 1.4.2 Software (AB Sciex). Quantitation of acylcarnitines, lipids and reducing mono- and oligosaccharides was accomplished by relating peak heights of the analytes to peak height of the chosen internal standard using the Met***IDQ*** Software (Biocrates Life Sciences AG). Met***IDQ*** contains all listed annotated metabolites with settings for validation. Quantitation of individual FAME (fatty acid methyl ester) was carried out with reference to the internal standard 18-methylnonadecanoic acid with the Agilent ChemStation Enhanced Data Analysis Software. The API4000 QTRAP was controlled using Analyst 1.4.2.

Concentrations of all analysed metabolites were corrected for natural isotope distribution using algorithms developed by Biocrates and implemented in the Met***IDQ*** software suite [20] and reported in µM units.

#### Proteome analysis

Urine samples were prepared as described in [7]. Briefly, a 0.7 mL aliquot stored urine was thawed and diluted with 0.7 mL 2 M urea, 10 mM NH_4_OH containing 0.02% SDS. Samples were filtered using Centrisart ultracentrifugation filter devices (20 kDa cut-off; Sartorius, Goettingen, Germany) at 3,000 g until 1.1 mL of filtrate was obtained. Subsequently, filtrate was desalted using PD-10 column (GE Healthcare, Sweden) equilibrated in 0.01% NH_4_OH OH in HPLC-grade water. Finally, samples were lyophilised and stored at 4°C prior analysis. The proteomics technique used was CE-MS. Shortly before CE-MS analysis, lyophilisates were re-suspended in HPLC-grade water to a final protein concentration of 0.8 mg/mL checked by BCA assay (Interchim, Montlucon, France). CE-MS analysis was performed as described [7], [8], [21]. The average recovery of sample in the preparation procedure was, ∼85% and the limit of detection was, ∼1 fmol. Mass resolution was above 8,000 Da enabling resolution of monoisotopic mass signals for z≤6. After charge deconvolution, mass accuracy was, <25 ppm for monoisotopic resolution and, <100 ppm for unresolved peaks (z.6). The analytical precision of the platform was assessed extensively [7], [21], [22].

### Proteomics data processing

Mass spectral peaks representing identical molecules at different charge states were deconvoluted into single masses using MosaiquesVisu software [23]. Only signals with z>1 observed in a minimum of 3 consecutive spectra with a signal-to-noise ratio of at least 4 were considered. Reference signals of 1770 urinary polypeptides were used for CE-time calibration by locally weighted regression. For normalisation of analytical and urine dilution variances, signal intensities were normalised relative to 29 ‘‘housekeeping’’ peptides [21], [24]. The obtained peak lists characterise each polypeptide by its molecular mass [Da], normalised CE migration time [min] and normalised signal intensity. All detected peptides were deposited, matched, and annotated in a Microsoft SQL database allowing further statistical analysis [25]. For clustering, peptides in different samples were considered identical if mass deviation was <50 ppm. CE migration time was controlled to be below 0.35 minutes after calibration. All data of the proteomic and metabolomic analyses were included in Table S2 and Table S3 in File S1.

### Statistical analysis and development of high dimensional classifiers

For biomarker discovery, statistical analysis was performed by the use of Wilcoxon rank sum test to calculate the p-values. Only biomarkers that were found at a 70% frequency in either case or control group were examined. The false discovery rate adjustments of Benjamini-Hochberg [26] were employed to correct for multiple testing. A p-value less than 0.05 was considered to be statistically significant. MosaCluster (version 1.7.0) was used to build a classifier based on support vector machine (SVM) that allows the classification of samples in the high dimensional data space [27], [28]. MosaCluster calculated classification scores based on the amplitudes of the CKD biomarkers. Classification is performed by determining the Euclidian distance (defined as the SVM classification score) of the vector to a maximal margin hyperplane. The SVM classifier uses the log transformed intensities of x features (peptides or metabolites) as coordinates in a x-dimensional space. It then builds a x-1 dimensional hyperplane that spans this space by performing a quadratic programming optimisation of a Lagrangian using the training labels only while allowing for samples to lie on the wrong side of the plane. For such mistakes in classification the SVM introduces a cost parameter C. Because non separable problems in low dimensions may be separable in higher dimensions the SVM uses the so called Kernel-trick to transform the samples to a higher dimensional space. MosaCluster uses the standard radial basis functions as kernel. These functions are just Gaussians with the parameter gamma controlling their width. The optimal parameters C and gamma are found via e.g. leave one out cross validation error estimation. There are generally implemented in SVMs in all popular data mining software, particularly the kernlab cran contributed R package is a versatile tool for building SVM based-classifiers [29]. After identification of significant biomarkers and generation of different classifiers, they were assessed in a test set to check their performance.

### Correlation of CKD classifiers with eGFR

After biomarker identification using the training set, CKD molecular classifiers were developed and their performance was assessed. Individual CKD classifier scores were correlated with eGFR at baseline and follow-up eGFR was used to predict the progression of the renal function. The test set of 29 patients was used in multiple correlation analyses using the classification scores of the different classifiers with baseline eGFR. For the correlation analysis with the follow-up eGFR after a period of 2.8±0.8 years, data from 43 out of 49 patients were available. The analysis was performed using MedCalc version 8.2.1.0 (MedCalc Software, Mariakerke, Belgium).

## Results

Urine and plasma samples obtained from patients representing different stages of CKD were divided into two cohorts: a training set (*n* = 20; Table 1) for biomarker identification and generation of CKD classifiers and a test set (*n* = 29; Table 1) to asses the classifier performance. The training set defined according to eGFR measurements included a “mild CKD” group (59.9±16.5 mL/min/1.73 m^2^, mean ± SD; *n* = 10) with patients between mild to moderate CKD and a second group named “advanced CKD” (8.9±4.5 mL/min/1.73 m^2^, mean ± SD; *n* = 10) with patients in advanced CKD that were matched for demographic and clinical data (Table 1). Follow-up data were obtained after 2.8±0.8 years. In the training set, the mean eGFR progressed to 61.2±26.2 mL/min/1.73 m^2^ (mean ± SD; Table 1) in the “mild CKD” group and to 8.7±3.1 mL/min/1.73 m^2^ (mean ± SD; Table 1) in the “advanced CKD” group.

### Metabolomic and proteomic biomarkers in urine and plasma

**Figure 1:**
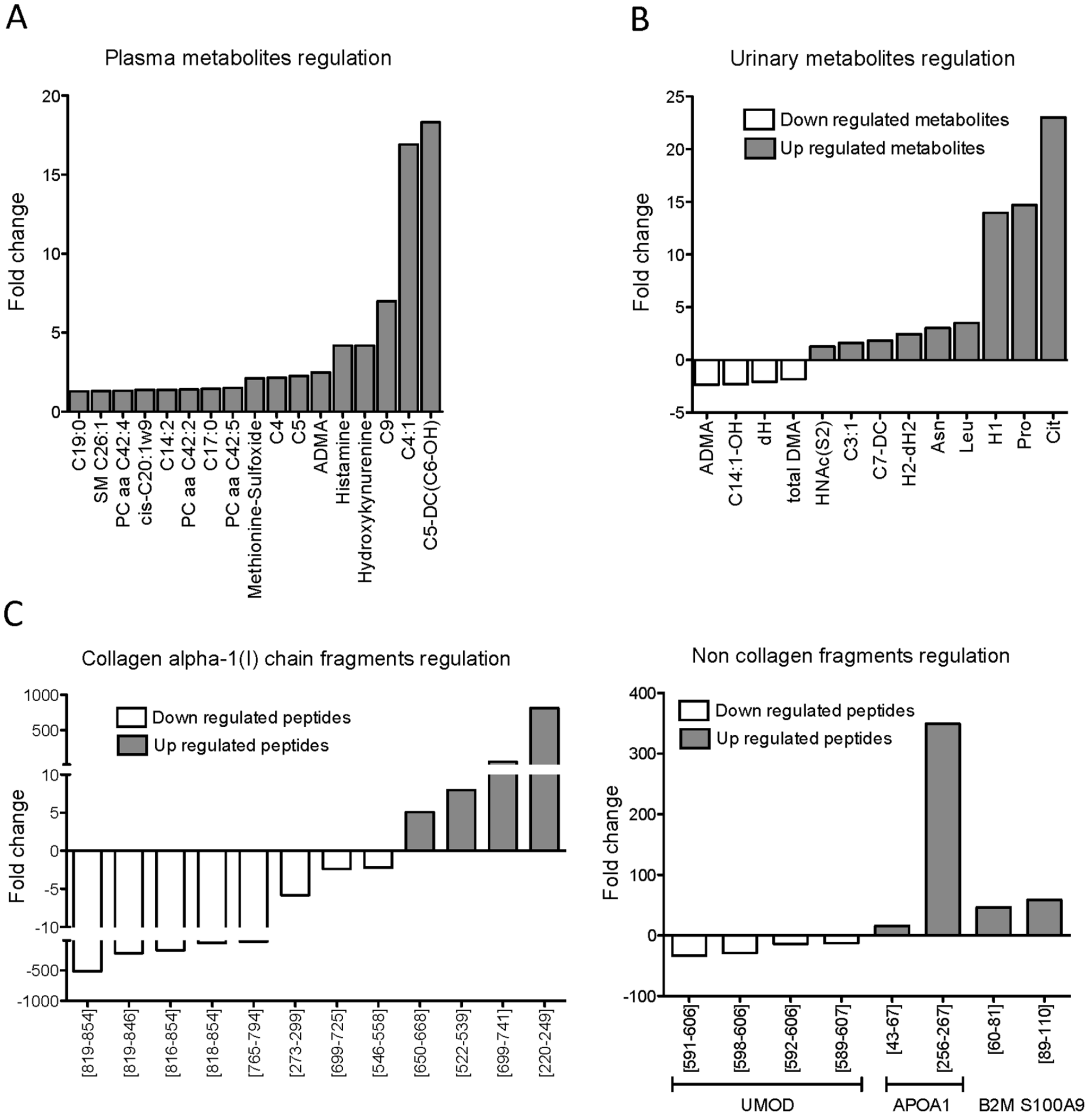
Regulation of metabolites and peptides. The fold changes of metabolites and peptides “mild CKD” vs. “advanced CKD”. A. Plasma metabolites. B. Urinary metabolites. C. Urinary peptides. C19∶0: Nonadecanoic acid. SM C26∶1: Sphingomyelin with acyl residue sum C26∶1. PC aa C42∶4: Phosphatidylcholine with acyl-alkyl residue sum C42∶4. C14∶2: Tetradecadienoylcarnitine. cis-C20∶1w9: cis-11-Eicosenoic acid. PC aa C42∶4: Phosphatidylcholine with acyl-alkyl residue sum C42∶4. C17∶0: Heptadecanoic acid. PC aa C42∶5: Phosphatidylcholine with acyl-alkyl residue sum C42∶5. C4: Nonanoylcarnitine. C5: Isovalerylcarnitine. ADMA: Asymmetric dimethylarginine. Total DMA: Total dimethylarginine. C9: Nonanoylcarnitine. C4∶1: Butenoylcarnitine. C5-DC(C6-OH): Acylcarnitine. C14∶1-OH: 3-Hydroxytetradecenoylcarnitine. dH: Deoxyhexose. HNAc(S2): (N-acetylhexosamine)-disulfate. C3∶1: Propenoylcarnitine. C7-DC: Pimelylcarnitine. H2-dH2: Dihexose-dideoxyhexose. Asn: Asparagine. Leu: Leucine. H1: Hexose. Pro: Proline. Cit: Citrulline.

The statistical analysis resulted in the identification of 76 significant biomarkers with p<0.05 (Table S1 in File S1). The biomarkers included 30 metabolites comprising 17 plasma metabolites (Figure 1A) and 13 urinary metabolites (Figure 1B) and 46 peptides (Figure 1C). Serum creatinine, one of the significant metabolomic biomarker identified in plasma, was excluded, as it is the major driver in the calculation of the eGFR.

Among the most significant metabolites identified were asymmetric dimethylarginine (ADMA) and hydroxykynurenine (Figure 1B, 1C and Table S1 in File S1). ADMA was the only identified metabolite present in both plasma and urine (Figure 1B and 1C). While the concentrations of ADMA and acylcarnitines were higher in the plasma samples of “advanced CKD” compared to the “mild CKD” group, ADMA concentrations were lower in the urine of late CKD patients (Figure 1A and B). Of the 46 urinary peptides to be significantly changed in CKD in this small study, 28 were collagen fragments with collagen type I alpha 1 being the most represented (Figure 1C and Table S1 in File S1). Eighteen additional non-collagen peptides were associated with CKD, including uromodulin, beta-2-microglobulin, apolipoprotein A-I, CD99 antigen and cadherin (Figure 1C and Table S1 in File S1). Most of the collagen type I (Figure 1C) and uromodulin fragments (Figure 1C) were in lower abundance in advanced CKD while beta-2-microglobulin, apolipoprotein AI and protein S100-A9 fragments were in higher abundance in advanced CKD (Figure 1C) in accordance with previous findings [7], [8].

Significant metabolite biomarkers associated with CKD were further combined into classifiers and assessed in the test set. Two different classifiers were established using metabolite biomarkers: one classifier incorporating the 17 metabolites from plasma only named MetaboP and another classifier based only on 13 urinary metabolite biomarkers named MetaboU. Likewise, a classifier based on proteomic traits alone was established with the 46 identified peptides in a classifier named Pept.

### Correlation of the biomarker based classifiers with baseline eGFR

**Figure 2:**
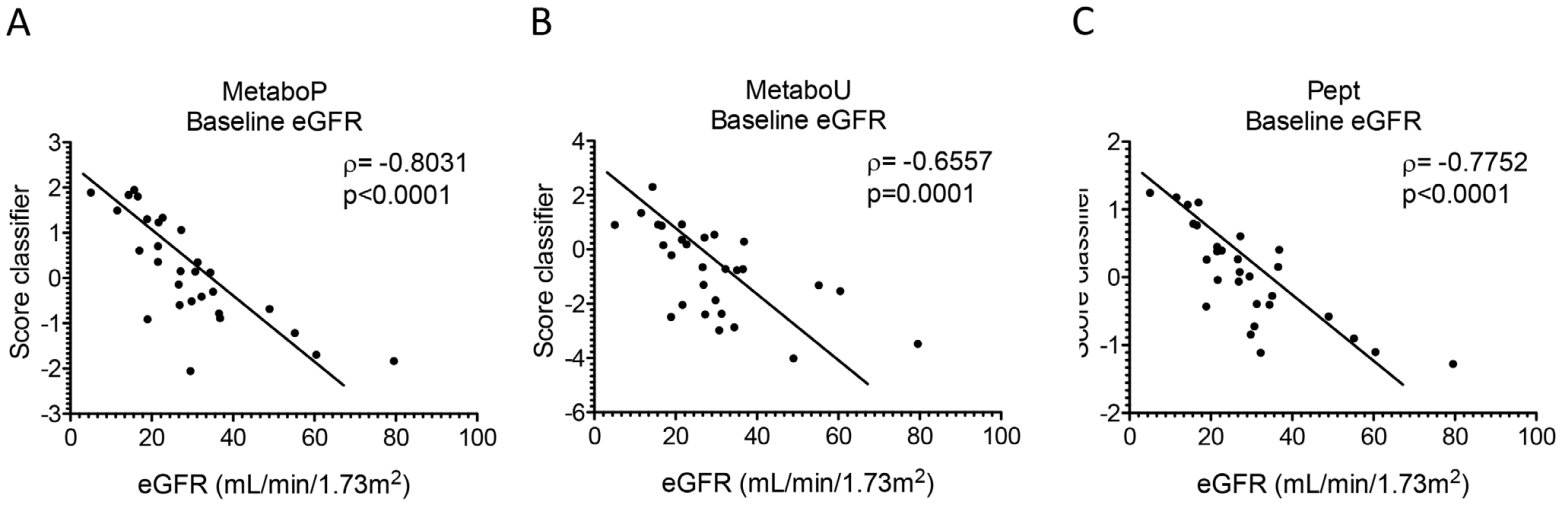
Correlation analysis of metabolomic and proteomic based classifier scores with baseline eGFR. The correlation analysis is performed by using the support vector machine classification scores obtained for the test set with baseline. A. Classifier MetaboP (plasma metabolites) ρ = −0.8031 and p<0.0001. B. Classifier MetaboU (urinary metabolites) ρ = −0.6557 and p = 0.0001. C. Classifier Pept (urinary peptides) ρ = −0.7752 and p<0.0001.

To assess the performance of each classifier at characterising the renal function, a correlation analysis based on the baseline eGFR was performed. The three classifiers MetaboP, MetaboU and Pept were significantly correlated with baseline eGFR (ρ = −0.8031, p<0.0001, Figure 2A; ρ = −0.6557, p = 0.0001, Figure 2B, and 0.7752, p<0.0001, Figure 2C, respectively). Individual comparison of Pept with MetaboU and MetaboP was not significant (p = 0.3712 and p = 0.7895, respectively).

### Assessment of the biomarker based classifiers in predicting future eGFR

**Figure 3:**
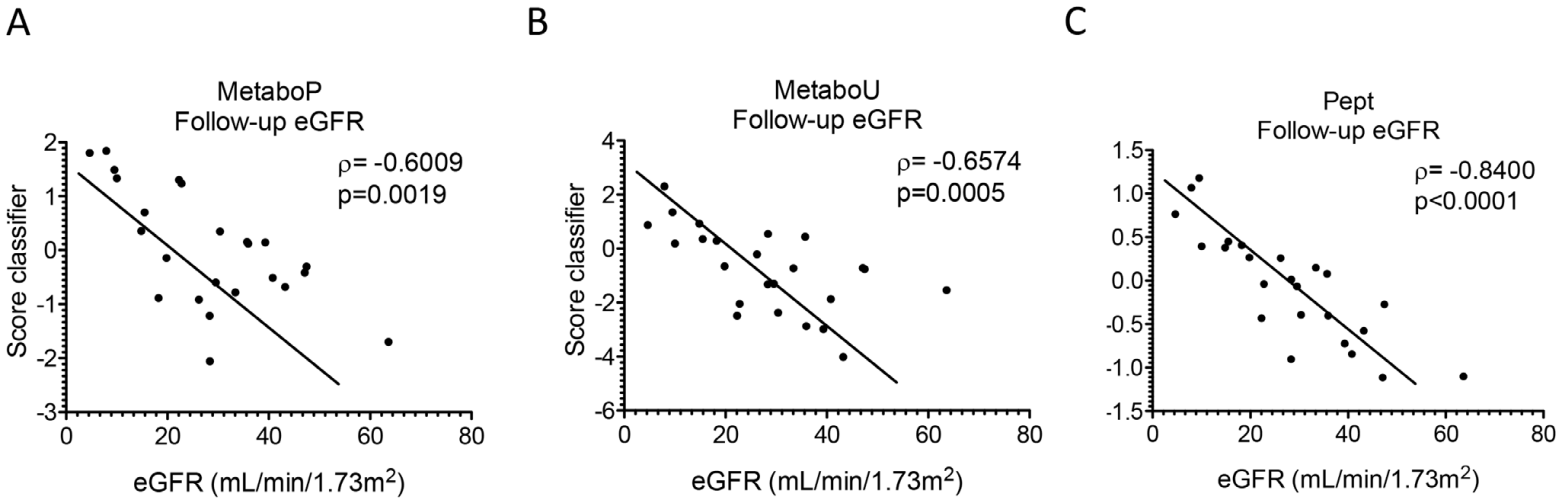
Correlation analysis of metabolomic and proteomic based classifier scores with follow-up eGFR. The correlation analysis is performed by using the support vector machine classification scores obtained for the test set with follow-up eGFR. A. Classifier MetaboP (plasma metabolites) ρ = −0.6009 and p = 0.0019. B. Classifier MetaboU (urinary metabolites) ρ = −0.6574 and p = 0.0005. C. Classifier Pept (urinary peptides) ρ = −0.8400 and p<0.0001.

The performance of above-mentioned CKD classifiers at predicting the progression of renal function was investigated using the follow-up data from the test set. The classifier MetaboP was significantly correlated with follow-up eGFR (ρ =  −0.6009, p = 0.0019, Figure 3A) and the classifier MetaboU also show a significant correlation (ρ = −0.6574, p = 0.0005, Figure 3B). The urinary peptide-based classifier was significantly correlated with the follow-up eGFR (ρ = −0.8400, p<0.0001, Figure 3C). The individual comparison of Pept with MetaboU and MetaboP (p = 0.1606 and p = 0.0879, respectively) again demonstrated no significant difference between the classifiers.

### Development of a classifier using combination of metabolomic and proteomic biomarkers

**Figure 4:**
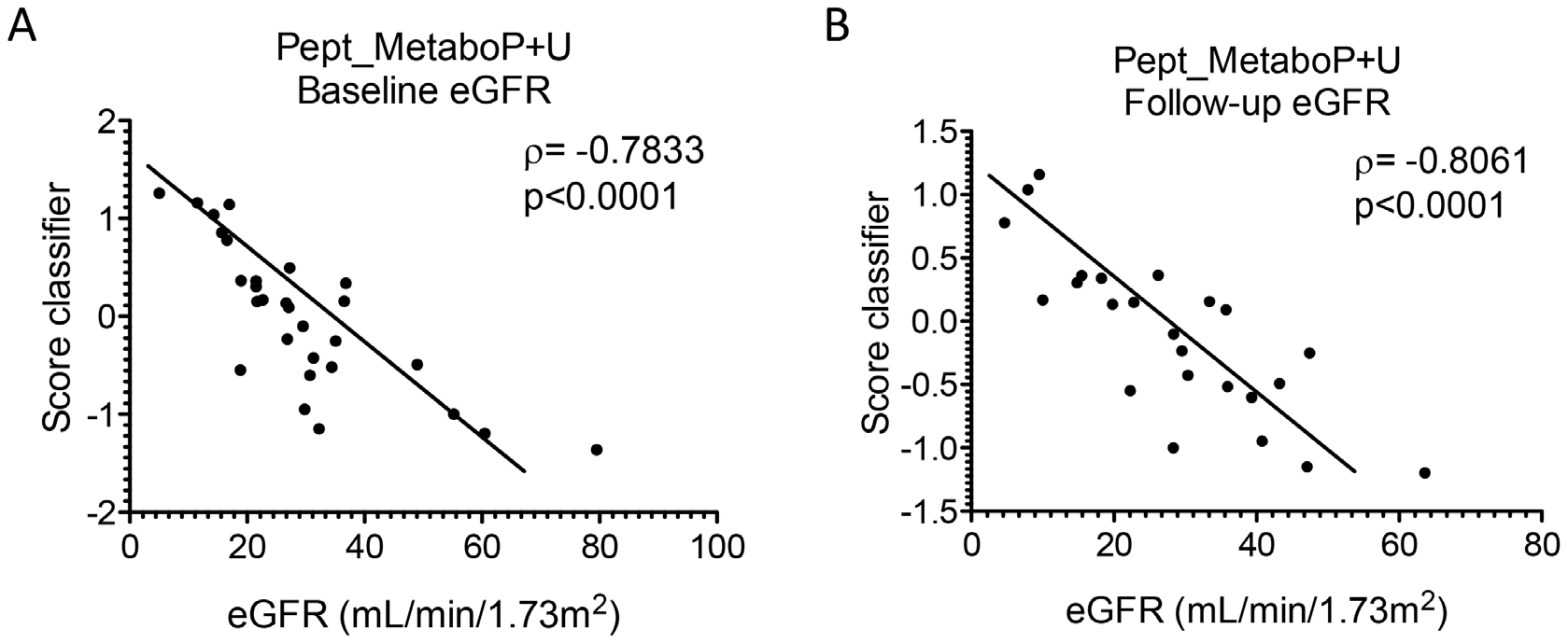
Correlation analysis of a combined proteomics and metabolomics based classifier with baseline or follow-up eGFR. A. Classifier Pept_MetaboP (urinary peptides and plasma metabolites) with baseline eGFR ρ = −0.7833 and p<0.0001. B. Classifier Pept_MetaboP with follow-up eGFR ρ = −0.8061 and p<0.0001.

To assess the potential of combining metabolomics and proteomics data, all identified biomarkers including 17 plasma metabolites, 13 urinary metabolites and 46 urinary peptides were unified in one classifier named Pept_MetaboP+U. In the test set, the classifier Pept_MetaboP+U showed a significant correlation at baseline eGFR with a correlation coefficient of ρ = −0.7833 (p<0.0001, Figure 4A). The comparison of correlation coefficients of Pept_MetaboP+U with MetaboU and MetaboP with baseline eGFR (p = 0.3328 and p = 0.8472, respectively) demonstrated no significant difference. Similar observations were made between Pept_MetaboP+U and Pept at baseline (p = 0.9407).

The classifier Pept_MetaboP+U also revealed a significant association with follow-up eGFR with a correlation coefficient of ρ = −0.8061 (p<0.0001, Figure 4B). The comparison of correlation coefficients of Pept_MetaboP+U with MetaboU and MetaboP at follow-up (p = 0.2885 and p = 0.1723, respectively) depicted no significant difference and these observations were also made between Pept_MetaboP+U and Pept (p = 0.7327).

## Discussion

The aim of the present study was to investigate the value of proteomics and metabolomics in assessing renal function, and to assess if combining metabolomic and proteomic approaches in one comprehensive biomarker-based classifier for CKD may be advantageous. We investigated the value of these molecular markers in a cross sectional design, and their performance in the prediction of the renal function decline.

Proteomics [7]–[9] and metabolomics [10]–[12] have already demonstrated value in classifying CKD patients. However, the diagnostic potential of the combination of both approaches has not been investigated so far. In our study, we examined samples from 49 patients at different stages of CKD. Urine samples were analysed employing proteomics, and urine and plasma samples were analysed using metabolomics. We identified a panel of 30 metabolites (17 plasma and 13 urinary metabolites) significantly different when comparing a training set of patients with early and with advanced stage CKD. In the same training set 46 peptides also demonstrated significantly different distribution. We combined these potential biomarkers in different classifiers and then performed correlation analyses with the baseline and follow-up eGFR in an independent test set. All three classifiers, plasma metabolite-based (MetaboP) urinary metabolite-based (MetaboU), and urinary peptide-based (Pept) correlated very well with eGFR, with no significant difference between them. Thus, the plasma and urinary metabolite and the urinary peptide-based classifiers individually were identified as effective tools associated with CKD.

The prognostic value of the classifiers was assessed based on the correlation with the follow-up data. The metabolite and peptide-based classifiers individually showed good performances in the prediction of future renal function. Although all classifiers performed equally well there seemed to be a tendency for the urinary peptide-based classifier to performed better in the prognostic evaluation than MetaboU and MetaboP (p = 0.1606 and p = 0.0879, respectively). However, a larger sample size would be required to investigate if this difference is in fact significant.

The results indicate that urinary and plasma metabolites and urinary peptides may provide similar information in the assessment of CKD. However, urinary peptides may demonstrate superior performance in a larger study [6].

An advantage of this study is that samples from patients representing all stages of CKD were included, which enabled identification of potential biomarkers representing the entire range of changes occurring throughout CKD progression with good confidence.

The combination of urinary peptide, urinary metabolite and plasma metabolite biomarkers in a classifier (Pept_MetaboP+U) showed a good correlation performance with eGFR at baseline (ρ = −0.7833, p<0.0001) and follow-up (ρ = −0.8061, p<0.0001). However, the comparison of single traits classifiers with the combined classifier showed no significant improvement suggesting that the combination of proteomics and metabolomics was not of an added value in our study.

In the current study 46% of the peptides and 26% of the metabolites identified were also previously reported [7], [30] (see Table S1 in File S1). The limited coverage of the peptides is due to differences in the study design as mild and advanced CKD patients were compared to enable identification of good confidence biomarkers instead of comparing between healthy and CKD patients. In the case of the metabolites only amino acids were investigated in the earlier study whereas we analysed amino acids, acylcarnitines, sphingomyelins, phosphatidylcholines, hexose (glucose), and biogenic amines. Besides mild versus advanced CKD detection and prediction of progression, the identified peptides as well as metabolites could potentially provide insight into the pathology of CKD. Most of collagen peptide fragments, representing the majority of detected urinary peptides, were reduced in patients with advanced CKD, which is in good accordance with previous studies [31], [32]. We hypothesise that this observation may mirror alterations in the extracellular matrix (ECM) turnover and fibrosis [33]. Renal fibrosis is one of the key features of CKD [34] and is characterised by ECM accumulation as a result of both, increased synthesis and reduced degradation of ECM proteins [35], [36]. Reduced abundance of urinary collagen fragments in CKD patients might thus reflect decreased ECM turnover. Renal fibrosis associated with CKD is the ultimate end-point of a cascade of events, including inflammation [37]. The observed elevation of protein S100-A9, a pro-inflammatory protein that promotes the migration of phagocytes [38] supports the presence of inflammatory processes. Urinary levels of uromodulin were also reduced which is in accordance with the literature as decreased uromodulin levels are associated with interstitial fibrosis or tubular atrophy [32].

In regard to metabolites, we observed increases of ADMA, hydroxykynurenine, and acylcarnitine levels in the plasma and a decrease of ADMA in the urine that significantly correlated with a decrease in the eGFR.

The observed changes in ADMA levels are consistent with previous observations in early and late stage CKD patients [39]–[42]. In one of these studies, it was shown that plasma and urinary levels of ADMA could be used to determine the CKD stage as plasma accumulation and lower urinary excretion pointed towards advanced CKD stages [43]. ADMA is a metabolite that inhibits nitric oxide synthase, an enzyme converting L-arginine to L-citrulline and nitric oxide (NO) [44]. Impaired generation of NO by accumulation of ADMA contributes to hypertension and in turn cardiac and renal dysfunction [45], [46].

The accumulation of various acylcarnitines in the plasma likely depicts impaired clearance due to chronic kidney dysfunction, which is consistent with recent observations [47]. Besides its function in fatty acid beta oxidation, L-carnitine modulates acyl-CoA levels through esterification to acylcarnitines, thus preventing the accumulation of acyl-CoAs generated in excess in renal failure [48], [49]. Excess acyl-CoAs may contribute to renal and cardiac lipotoxicity [50]–[52]. Hence, the resulting excess acylcarnitines normally are filtered in the glomerulus and undergo only limited renal tubular reabsorption compared to free L-carnitine [53]–[56].

Hydroxykynurenine is part of the kynurenine pathway and generated as a result of tryptophan degradation [57]. Increased plasma levels of hydroxykynurenine have previously been reported to be associated with advanced stage CKD [58], [59]. The association of hydroxykynurenine with CKD is not very well understood. A hypothesis was presented that accumulation of hydroxykynurenine could be a result of oxidative stress leading to impaired renal function [60]. In addition, phosphatidylcholine diacyl C42∶5 increased in the plasma of patients with severe renal impairment. Phosphatidylcholine diacyl C42∶5-to-phosphatidylcholine acylalkyl C36∶0 ratios were found to be associated with the loss of eGFR in CKD patients in a longitudinal study [10].

In conclusion, we could demonstrate in this study the feasibility of combining proteomic and metabolomic approaches in the prediction of renal function. However, we could not demonstrate an advantage of combining these different omics traits. In contrast, our data indicate that essentially a solely urinary peptide, urinary metabolite and plasma metabolite-based approaches may be sufficient to predict renal function and that combining metabolomics and proteomics may not provide significant added value. The results also suggest that urinary peptides may be superior in predicting renal function decline. However, these results are based on a small cohort and need to be further reproduced in large independent cohorts. The results are valid only in the context of CKD, and the same concept may well be found advantageous in the diagnosis of other diseases like coronary artery disease.

## Supporting Information

File S1 **Supporting tables.** Table S1, List of significantly identified biomarkers between “Mild CKD vs. Advanced CKD” at baseline. 46 peptides, 17 plasma metabolites and 13 urinary metabolites derived from the training set of mild and advanced CKD at baseline. The statistical and correlation analysis of each biomarker with the eGFR were performed at baseline. In the table, the biomarker marker ID, name, type, source, the Spearman's rank correlation coefficient and its p-values, the p-values from the statistical analysis (Benjamini Hochberg), the comparison with biomarkers identified in previous CKD studies were provided. In the “comparison with CKD studies” column, x indicates that the biomarker was previously identified. Table S2, Raw data of identified peptides at baseline. The raw data of all 46 peptides were provided at baseline for the 49 patients used in the study. Each column represents a patient and the patient ID goes from 1 to 49. Table S3, Raw data of identified metabolites at baseline. The raw data of all 30 metabolites (17 in the plasma and 13 in the urine) were provided at baseline for the 49 patients used in the study. Each column represents a patient and the patient ID goes from 1 to 49.
(XLS)
